# Operation for Perforation in Typhoid Fever

**Published:** 1903-01-24

**Authors:** 


### OPERATION FOR PERFORATION IN TYPHOID FEVER.

AFTER the depressing reports which have recently come to hand in regard to operations undertaken in cases of perforation of the bowel in typhoid fever, it is satisfactory to find at a recent meeting of the Clinical Society that Mr. Bowlby was able to relate a successful case. The patient, a boy aged 10 years, was admitted to St. Bartholomew's Hospital on the third day of an attack of typhoid fever. This was on September 1st. The attack was a severe one with relapse. On October 4th, two hours after the symptoms of perforation had occurred, the abdomen was opened in the middle line and at once free gas was found in the peritoneum. A considerable quantity of almost clear fluid welled up, but there was no large collection of fæcal matter. The intestines were so much distended that several coils had to be allowed to extrude. Starting from the cæcum Mr. Bowlby rapidly passed the small intestines through his hands till he found a perforation situated less than 2 feet from the ilio-cæcal valve. The bowel was not adherent nor could any induration be felt in its walls, but the peritoneum corresponding to a Peyer's patch was very red and injected. The opening in the intestine was very small, being not much larger than a pin's head. The bowel was wiped clean and the opening was closed by five Lembert's sutures of fine silk. The greater part of the incision was then closed, a glass drainage tube being placed in its lower end. The peritoneum was not douched but the intestines were carefully wiped clean and all fluid was mopped up from the pelvis and elsewhere with sponges. For several days the boy remained very ill. The morning after the operation the pulse was still 150, but there had been no sickness, and flatus had been passed. Various things happened to delay his recovery, but in the end he got quite well. Commenting upon this case Mr. Bowlby said that this and one other, which had also been one of perforation occurring late in the course of typhoid fever, were the only cases of the kind on which he had operated, and there could be no doubt that they represented two lives saved. He did not, however, regard them as at all typical cases of typhoid perforation, but rather as typical of a special class of case in which perforation occurs after the fever is practically over, and in which the general condition of the patient is otherwise relatively good. In such patients the symptoms of perforation are generally well marked and the diagnosis is readily arrived at. But in many cases of perforation the reverse is true, as when the accident occurs while the fever is yet high in patients who are more or less unconscious, in whom there may be no expression of pain, and who may have distended and tympanitic abdomens before perforation occurs. Many such patients are already at death's door, and often in addition to the perforation which has taken place there are numerous other areas where the intestine is so rotten that its giving way is only a question of time. In such cases recovery is not to be expected. Mr. Bowlby referred to cases which he had seen in South Africa, in which the question of operation had arisen, and in which on post-mortem examination the intestine was so matted and adherent to the peritoneum and mesentery that even after death it could with difficulty be unravelled, so that, although there was a perforation, it would not have been discovered by operation, even if the patient's condition has permitted one to be performed.

